# Ovarian Cancer Incidence Corrected for Oophorectomy

**DOI:** 10.3390/diagnostics7020019

**Published:** 2017-04-01

**Authors:** Lauren A. Baldwin, Quan Chen, Thomas C. Tucker, Connie G. White, Robert N. Ore, Bin Huang

**Affiliations:** 1 The Division of Gynecologic Oncology, Department of Obstetrics and Gynecology, The University of Kentucky College of Medicine, 800 Rose Street, 330 Whitney-Hendrickson Building, Lexington, KY 40536, USA; robert.ore@uky.edu; 2 Division of Cancer Biostatistics, College of Public Health & Biostatistics Shared Resource Facility, Markey Cancer Center, University of Kentucky, Lexington, KY 40506, USA; quan.chen@uky.edu (Q.C.); bhuang@kcr.uky.edu (B.H.); 3 Department of Epidemiology, College of Public Health & Kentucky Cancer Registry, Markey Cancer Center, University of Kentucky, Lexington, KY 40506, USA; tct@kcr.uky.edu; 4 Kentucky Department for Public Health, Frankfort, KY 40601, USA; Connie.White@ky.gov

**Keywords:** ovarian cancer, prevalence, incidence, oophorectomy, screening

## Abstract

Current reported incidence rates for ovarian cancer may significantly underestimate the true rate because of the inclusion of women in the calculations who are not at risk for ovarian cancer due to prior benign salpingo-oophorectomy (SO). We have considered prior SO to more realistically estimate risk for ovarian cancer. Kentucky Health Claims Data, International Classification of Disease 9 (ICD-9) codes, Current Procedure Terminology (CPT) codes, and Kentucky Behavioral Risk Factor Surveillance System (BRFSS) Data were used to identify women who have undergone SO in Kentucky, and these women were removed from the at-risk pool in order to re-assess incidence rates to more accurately represent ovarian cancer risk. The protective effect of SO on the population was determined on an annual basis for ages 5–80+ using data from the years 2009–2013. The corrected age-adjusted rates of ovarian cancer that considered SO ranged from 33% to 67% higher than age-adjusted rates from the standard population. Correction of incidence rates for ovarian cancer by accounting for women with prior SO gives a better understanding of risk for this disease faced by women. The rates of ovarian cancer were substantially higher when SO was taken into consideration than estimates from the standard population.

## 1. Introduction

Cancer incidence rates are calculated by dividing new primary cancer cases of a disease by the population at risk in the same time period adjusted by the US standard population [1]. This assessment has great importance clinically, especially for gynecologic oncology with regard to training a sufficient number of physician specialists. Cancer incidence rates help physicians and researchers assess risk levels, which can be used for public health and individual patient education, to prioritize prevention and research efforts, and to guide assessment of the cost and efficacy of cancer screening. Thus, the accuracy of this risk assessment is very important.

There is an inherent problem in the incidence calculation for some malignancies due to the inclusion of patients in the denominator who are not at risk for the disease [2]. In gynecologic oncology, this has been most thoroughly evaluated in the case of endometrial cancer and hysterectomy. Many women will undergo hysterectomy in their lifetime for a variety of benign conditions [3,4]. These women are not at risk for developing endometrial cancer after uterine removal and should not be included in the population at risk for incidence rate calculation. There have been multiple publications that have evaluated methods of correcting risk calculations for endometrial cancer. In 2012, Siegel et al. reported on age-adjusted, hysterectomy-corrected uterine cancer rates stratified by race and geography and found that failure to adjust rates for hysterectomy leads to distortion of racial and geographic patterns and underestimates disease burden [5].

There is less guidance in the literature concerning the impact of oophorectomy rates on ovarian cancer incidence. Many women have salpingo-oophorectomy (SO) performed alone due to benign ovarian disease, or have SO performed at the time of hysterectomy for benign conditions. There has been new evidence linking the origin of serous ovarian cancer to the fimbriated end of the fallopian tube [6,7,8,9,10,11,12,13,14,15,16,17]. During adnexal surgeries, the tube is usually removed concurrently with the ovary. Whether the pathogenesis for the most common type of ovarian cancer truly arises from the distal tube or the ovary itself, those who have undergone SO should have drastically reduced risk of this type of malignancy. This reduction has been demonstrated in high risk women with Breast Cancer Susceptibility (*BRCA)* mutations who undergo risk-reducing salpingo-oophorectomy and have dramatically lower risk of ovarian malignancy [16,17,18,19].

The current reported incidence of ovarian cancer from 2005–2009 is 12.7 per 100,000 women [20]. Incidence rates are higher in whites and the average age at diagnosis is 63 [20]. Lifetime risk of developing ovarian cancer in the United States is 1.4% [20]. In 2016, 22,280 new cases were expected and 14,240 deaths anticipated from this disease [21]. Ovarian cancer is the 5th leading cause of death from malignancy in women in this country due to the fact that the majority of cases are diagnosed at an advanced stage. Research into prevention and screening for ovarian cancer is hampered by this low prevalence, which negatively affects accurately estimating the positive predictive value for these tests. We hypothesize that when incidence rates are corrected for prior SO, incidence of this disease will be higher than in commonly reported statistics which currently underestimate the risk of ovarian cancer for women whose ovaries and/or tubes remain intact. 

## 2. Materials and Methods

### 2.1. Cancer Incidence Data

To calculate the cancer incidence rates, the most recent five-year ovary cancer cases diagnosed in years 2009–2013 from the Kentucky Cancer Registry (KCR) were extracted. Ovary cancer cases were defined as ICD-O-3 site codes C569 excluding ICD-O-3 histology codes 9050–9055, 9140, 9590–9992. Only invasive cancer cases were included for the analysis.

The KCR is a population-based registry, and has been awarded the highest level of certification by the North American Association of Central Cancer Registries for an objective evaluation of completeness, accuracy, and timeliness every year since 1997. The KCR is part of both the CDC National Program of Cancer Registries and the NCI Surveillance, Epidemiology, and End Results (SEER) program, which are considered among the most accurate and complete population-based cancer registries in the world. The KCR also links its database annually with the National Death Index (NDI) to capture the most accurate survival information. No new data was collected from subjects specifically for this study and no contact with any patients was required. All data was previously de-identified.

### 2.2. Kentucky Health Claims Data (KHCD)

In order to correctly calculate the age-adjusted rates for ovary cancer incidence, the underlying risk population needs to be modified to reflect the fact that women who had SO will have minimal risk of having ovarian cancer. To estimate the prevalence of women who had prior SO for years 2009–2013 in Kentucky, the Kentucky health claims data (KHCD) 2000–2014 data sets were acquired from the Office of Health Policy in the Kentucky Cabinet for Health and Family Services (KCHFS).

The KHCD data include hospital discharge reports from all Kentucky hospitals, Medicare provider-based entities and ambulatory facilities (http://lrc.ky.gov/KAR/900/007/030.htm). The data include in-patient and out-patient files containing de-identified individual records. Key elements, such as ICD-9 procedure codes, CPT codes, and demographics are included in the files. Age is presented in the format of age groups. 

### 2.3. Kentucky Behavioral Risk Factor Surveillance System (BRFSS) Data

The Behavioral Risk Factor Surveillance System (BRFSS) data is the annual telephone survey that collects state data related to health risk behavior, chronic health conditions, and use of preventive services for all 50 states, the District of Columbia, and three territories in the U.S (https://www.cdc.gov/brfss/). For women aged 18 and older, responses to the question “Have you had a hysterectomy?” are included. The data related to this question was used to estimate the prevalence of prior hysterectomy by age group. Since the hysterectomy question was presented every other year, the Kentucky BRFSS data 2008–2012 was acquired from the KCHFS to match the ovary cancer incidence data 2009–2013.

### 2.4. Estimating Oophorectomy Prevalence

To estimate the prevalence of prior SO for Kentucky women in 2009–2013, two approaches were used. The first method estimated the full SO prevalence rates directly from the KHCD data and the second method estimated the SO prevalence rates based on both BRFSS data and KHCD data.

In the first method, SO cases were identified by ICD-9 procedure codes and CPT codes from the KHCD data for the years 2000–2014. Since the KHCD data in 2000–2003 did not include age or CPT codes and 2014 data were beyond the study period, only data for years 2004–2013 were used for the data analysis. The combined counts of SO cases by year and age group from both in-patient and out-patient files were considered as the total SO incidence. The age groups in KHCD data were categorized as 0, 1–5, 6–10, …, 76–80, 81+ years. Statistical approaches to estimating prevalence from incidence data commonly involves mortality and survival data, and can be either parametric or non-parametric [22,23,24,25]. Counting Method, a non-parametric approach, was used to estimate prevalence of prior SO based on the SO incidence data from the KHCD [23]. This approach counts cases of ‘still alive’ individuals on the desired prevalence date while making adjustment based on the estimates of cases lost to follow-up. For example, the number of prevalence case in age i and calendar year j was estimated as

$$N_{ij}=\int_{0}^{i}I\left(t\right)S\left(t,i-t\right)dt$$

where I(t) is the number of incidence in age t, and S(t,i−t) is the survival probability from all causes from age t to i−t. Since the KHCD data do not include survival and mortality data, the US 2010 female life tables were used to estimate the survival probabilities from all causes in the specific years and age groups. Bridged life tables to match the age group defined in the KHCD were calculated from the complete US 2010 female life table [26]. Because no SO incidence data by age group were available prior to 2004, it was assumed that the incidence data prior to 2004 were same as in the average of 2004–2013. To understand the impact of the assumption, the same calculation was also done while assuming incidence data prior to 2004 was the same as in the year 2004 and the year 2012, as the highest count of prevalence was identified in year 2004 and the lowest in 2012. To reflect the fact that the US life expectancies have increased over time and that women with oophorectomy had lower life expectancies than the general population [27], the probability of survival estimates were lowered from values in the US life tables by 0.5% when calculating the complete prevalence rates for prior SO.

To validate the prevalence estimates from the first method, we also used the BRFSS data to estimate the prevalence rates. In previous published studies, prevalence rates of prior SO were estimated by multiplying the prevalence rates of hysterectomy from the BRFSS data by the proportion of hysterectomy incidences with bilateral oophorectomy [2]. Similarly, we calculated the ratio of SO vs. hysterectomy by age group from the KHCD data for years 2004–2013 and the weighted prevalence rates of prior hysterectomy by age group for those aged 20+ from the BRFSS data for 2008, 2010 and 2012. The prior SO prevalence estimates by age groups were the product of the ratio of SO vs. hysterectomy and the prevalence of prior hysterectomy from the BFRSS data.

### 2.5. Age-Adjusted Incidence Rates for Ovary Cancer

All age-adjusted rates were calculated based on the standard 2000 US population. To examine how the different formats of age groups in the background population impact age-adjusted rates, the traditional age-adjusted rates based on the 19 age groups in the standard Kentucky population were calculated along with the traditional age-adjusted rates based on the 18 age groups defined in the KHCD data. To calculate the corrected age-adjusted rates for ovary cancer, the standard Kentucky population data were corrected by deducting the number of women with SO derived through the prevalence estimates from the two approaches previously discussed. 

All analyses were done using SAS Statistical software version 9.4. SAS (SAS Institute, Cary, NC, USA) was also used to develop programs to calculate the complete prevalence rates from the KHCD data. Statistical tests were two sided with a *p*-value ≤ 0.05 used to identify statistical significance.

## 3. Results

**Figure 1:**
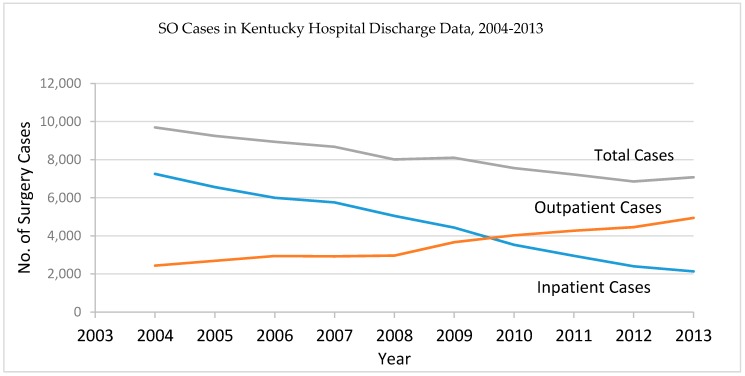
Trend of salpingo-oophorectomy (SO) cases from the Kentucky Hospital Discharge Data, 2004–2013.

There was a total of 81,359 SO cases identified from the KHDC data during 2004–2013 (Table 1). The highest frequencies were found in the age groups 41–45 and 46–50. Very few cases were found in women with ages younger than 20 or ages older than 80. The number of SO cases from the inpatient files had dropped steadily over the study period and the number of cases from the outpatient files had increased. The overall SO cases had consistently dropped since 2003 (Figure 1).

Using the Counting Method, estimates of annual prevalence counts and rates by age group for the years 2009 to 2013 were calculated from the KHCD. Only results from the years 2009 and 2013 based on the assumption that SO incidences prior 2004 are same as the average in 2004–2013 are shown in Table 2. The results based on the assumptions that SO incidences prior 2004 are same as in 2004 or 2012 can be found in Tables S1 and S2. The prevalence rates increased by age and peaked at the oldest age groups of 76–80 and 81+. Because of the decreasing trend of SO cases, the prevalence rates had dropped from year 2009 to 2013. For example, the rates dropped from 27.4% to 24.4% in the age group 61–65 and from 42.1% to 38.1% in the age group 71–75 (Table 2).

In Table 3, hysterectomy prevalence rates by age group were calculated from the BRFSS data for 2008, 2010, and 2012. The highest rates appeared in the age group 76–80. Ratios of SO vs. hysterectomy from the KHCD data varied from 65% to 103% by age group. The prior SO prevalence rates modified from the hysterectomy rates from the BRFSS data peaked in the age group 66–70 (50.7%) and were considerably smaller in the age group 81+ (42.5%) compared to the prevalence rates from the Counting Method.

A total of 1403 invasive ovary cancer cases for years 2009–2013 were extracted from the KCR database. The age-adjusted rates from the standard Kentucky population show the rates 10.7 per 100,000 (95% Confidence Interval (CI) 10.2–11.3) for all ages (Table 4). To match the age groups defined in the KHCD data, the age adjusted rates based on the standard Kentucky population with modified age groups were also calculated. The corrected age-adjusted rates from adjusting the population under risk based on the prevalence estimates of prior SO from the KHCD data were 15.5 (95% CI 14.7–16.3) per 100,000 assuming SO incidences prior 2004 were the same as the average of the incidence in the years 2004–2013, 16.9 (95% CI 16.0–17.8) per 100,000 assuming the SO incidences prior to 2004 were the same as in 2004 (highest incidence), and 14.3 (95% CI 13.6–15.1) per 100,000 assuming the SO incidences prior to 2004 were the same as in 2012 (lowest incidence). The corrected age-adjusted rate from the BRFSS prevalence estimates of SO was 17.7 (95% CI 16.8–18.7), which is higher than the highest estimates from the KHCD data (16.9 per 100,000). Overall, risk population adjusted SO age-adjusted rates ranged from 33% to 65% higher than the rates from the standard population. We also included the age-specific rates for ovary cancer by various approaches in Table S3.

## 4. Discussion

In the efforts reported here, the rates of ovarian cancer were 33% to 65% higher when prior SO was taken into consideration than estimates from the standard population. Due to the limitation of data availability, the risk-population adjusted prior SO rates have rarely been calculated previously. In the current study, we used the KHCD data and the Counting Method, a modern statistical approach, to estimate the prior SO prevalence rates based on various assumptions and the risk-population adjusted SO rates. We also estimated the SO rates using estimated SO prevalence rates from the BRFSS data. The prevalence rates of prior SO from the Counting Method and the BRFSS data are different because of various assumptions and different data sources, hence leading to the variation of the risk-population adjusted SO rates. The results demonstrate the challenge to correctly estimate the rates because of the data limitations.

Compared to previous published studies with only one type of estimate [2], our study is able to provide a range of estimates that gives a more comprehensive view of the estimates. It is possible that the 0.5% survival deduction of probability of annul survival from the standard US life table was too harsh and caused the lower estimates of SO prevalence rates compared to the estimates from the BRFSS data. Using the ratio of SO vs. hysterectomy from the KHCD data to estimate SO prevalence rates from the BRFSS was likely biased as the ratio was based on incidence data, not prevalence data.

Ovarian cancer remains the deadliest gynecologic malignancy in the United States, being the 5th most common cause of cancer death in women. Over 14,000 deaths from ovarian cancer are expected for the US in 2016 [20]. Despite advances in operative care and chemotherapy, including the recent use of targeted agents for this disease, overall survival remains poor [20,28,29,30]. While ongoing research efforts continue to search for better treatments with which to combat this disease, another approach to improve survival is through screening and earlier detection of disease. The majority of ovarian cancer cases are diagnosed at advanced stage prior to the onset of symptoms. Pelvic exam has been shown to have limited value in detecting ovarian abnormalities, especially in postmenopausal and obese women [31]. Only 15% of cases are confined to the ovary at the time of diagnosis [32]. However, survival is much improved for women who are diagnosed at an early stage [26]. Therefore, efforts to increase the detection of early stage disease have a potential to greatly impact survival. Estimates that reveal the true risk of ovarian cancer will support efforts to screen for early stage disease. 

Screening for malignancy has been highly effective for other common malignancies such as breast and cervix cancers [33,34]. Ovarian cancer meets criteria as a disease that could benefit from effective screening since it is the 5th leading cause of cancer mortality in women with proven improved survival when diagnosed at an earlier stage [20,26]. Screening has been studied in ovarian cancer, most commonly with serum Ca125 levels and transvaginal ultrasound (TVUS) or a combination of the two [35,36]. There have been four major trials that have evaluated ovarian cancer screening. The first of these is the prostate, lung, colorectal, and ovarian (PLCO) trial, which showed no benefit to screening [37]. There was a multicenter prospective randomized trial in Japan that compared screening with pelvic exam, serum Ca125, and ultrasound to routine care and saw an increase in the rate of optimal debulking in the screen detected cancers [38]. Optimal debulking has a known association with improved survival in ovarian cancer [39]. The University of Kentucky Ovarian Cancer Screening Trial (UKOCST) has been in progress since 1987 [40,41]. Over 45,000 women have been screened to date with TVUS. Detection of 47 ovarian cancers has been reported by the UKOCST and these women have improved five-year survival and are more likely to be early stage than women with clinically detected cancers [29,34,42]. Most recently, the results of the UKCTOCS randomized trial were published in the Lancet and have shown a survival benefit for screening [30].

Taken together, the available data from these four trials suggests screening works to detect disease at an earlier stage, which leads to improved survival. However, one of the most common criticisms of screening and the studies that have evaluated it is the lower positive predictive values, which are likely driven by the lower prevalence of this disease. Statistical calculations for predictive values vary greatly depending on the prevalence of the disease being studied, unlike sensitivity and specificity of a test, which remain constant. Thus, a test with inherently good sensitivity and specificity can be brought to improved predictive ability by narrowing the screening population to a high-risk group for which the prevalence is high.

One way to narrow threat risk population for ovarian cancer is by focusing on ages at which incidence is high. This has been commonly applied in previous screening trials and the results of the work reported here confirm the importance of age. In the present study, ovarian cancer incidence is highest for women over age 75, while the rate of hysterectomy peaks at age 65. Age continues to be one of the most important risk factors for ovarian cancer.

Given the importance of correct incidence to predictive calculations for screening programs and epidemiologic risk assessment, an accurate calculation of incidence is critical. An accurate assessment of risk is more easily determined in some diseases than others. If all subjects are at risk, then the calculation is a straight forward division of those diagnosed with disease by those at risk. This is not so clear in all diseases, however. For example, surgical interventions for unrelated problems can reduce the at-risk pool for certain disease sites. This has been demonstrated in the literature regarding endometrial cancer [5,43]. Correcting risk rates for endometrial cancer involves reducing threat risk pool (or denominator of the calculation) by removing those women who have undergone prior hysterectomy for a benign condition. Ignoring hysterectomy underestimates the risk for women who have not undergone that procedure and has also been shown to distort data regarding the distribution of disease [44]. Hysterectomy has recently been declining in nationwide statistics for the US, but remains one of the most common procedures performed in this country today, which alters the epidemiology significantly for uterine derived cancer risk [38]. Approximately 600,000 hysterectomies are performed each year in the United States, and around a third of all women have had the procedure by the time they turn 60 [45,46,47].

Salpingo-oophorectomy (SO) is even more difficult to quantify than hysterectomy. Many women elect to have their ovaries and tubes removed at the time of a hysterectomy that is performed for a variety of reasons related to primary uterine pathologies. Additionally, many women undergo bilateral SO either separate from a hysterectomy or at some time after a hysterectomy has been performed for a wide variety of primary ovarian or other conditions, many of which are benign. These include endometriosis, non-cancerous ovarian cysts or masses, risk reduction for genetic conditions, and for hormone reduction in breast cancer patients. The overall trend for SO in the US has been on the decline [48]. This decline coincides with the similar decline in hysterectomy rate. This decline may also be a result of data showing that surgical menopause prior to age 50 in women who never used estrogen is associated with increased all causes mortality [49]. Despite this, the rates of SO remain significant [42].

Given the robust number of women who have undergone SO, risk of ovarian cancer is greatly reduced for these women and importantly alters the epidemiology of risk for malignancy at this site on a population level. There are some important caveats to this reduction. Serous peritoneal cancers behave nearly identically to ovarian cancer and the risk for these cancers is unlikely to be altered by SO [50]. It should be noted that peritoneal cancer is quite rare. The protective effect of SO is illustrated in high risk women who have undergone prophylactic SO for BRCA mutation, and have achieved a drastically reduced risk of serous malignancy [16,17,18,19]. In one study, the relative risk of ovarian, fallopian tube or peritoneal carcinoma in women with known BRCA mutations after risk reducing bilateral SO was 0.04 (95% CI 0.01–0.16) [18]. Recent literature supports two separate types of ovarian malignancy, with separate pathogenesis [6,7,8,9,10,11,12,13,14,15,16,17]. Type 1 tumors are generally considered low grade malignancies that arise from the epithelium of the ovaries. Type 2 cancers generally include high grade serous malignancies that are felt to arise from the distal, fimbriated end of the fallopian tube. The fallopian tube is generally removed with the ipsilateral ovary in most procedures that are performed—few indications, if any, would preserve the tube if the ovary is being removed. Thus, protection from Type 2 ovarian malignancy is gained from ovarian removal in most cases since the tube is removed concomitantly (i.e., a salpingo-oophorectomy is typically performed rather than an oophorectomy alone).

An additional consideration is that there is a rising trend in bilateral salpingectomy rather than bilateral salpingo-oophorectomy, which allows ovarian preservation while still potentially reducing cancer risks [42]. The degree to which this procedure is as protective as SO has yet to be determined. The current study takes into account women having SO but not salpingectomy alone. An argument can be made to include these patients for future studies, which has the potential to further correct the underestimation of the prevalence of ovarian cancer for women who retain all portions of their adnexa.

Overall, the SO rate nationally has been reported to be declining. However, it is still common for women to undergo this procedure and greater than 40% of women still undergo bilateral SO at the time of hysterectomy [42,51]. This significant rate needs to be taken into account for estimating accurate ovarian cancer incidence. Failing to recognize and account for the population of women no longer at risk underestimates the incidence for the rest of the population. This was the driving motivation for the current study and the results confirm that incidence rates need to take surgical procedures into account. Incidence is certainly higher by all methods used for calculation in this study once SO was taken into account. This was true both with overall incidence across ages, as well as age-adjusted groups. 

There is inherent difficulty in establishing the overall risk associated with SO. Prior studies have shown an overall mortality disadvantage for women who undergo premenopausal oophorectomy—prior to age 50—and never used estrogen therapy [43]. This decrease in lifespan may be attributed to changes in cardiovascular health and other important roles provided through the hormonal functions of the ovary. How this risk quantitatively translates to changes in expected lifespan on an annual statistical level is unclear. This study estimated decreased survival of 0.5% annually for women who had undergone premenopausal SO. However, these are estimates and the true annual change in expected survival is unknown. Even taking this into account though, all calculations still show that incidence is underestimated if SO is not taken into account when evaluating ovarian cancer.

The strengths of this study include the use of population level data and novel statistical evaluation to correct risk assessments in an important way for women with regard to ovarian cancer. A couple of limitations are worth noting. Most notably, this study is limited by the dependence on CPT and ICD codes for diagnosis. Actual operative reports or pathology reports were not available for confirmation of procedure performed. This introduces the possibility of inappropriate coding leading to incorrect inclusion (or exclusion) of patients in the analysis. In addition, newer ICD codes are more specific in that bilateral procedures are noted and were isolated for inclusion. This is important as unilateral procedures would not be expected to confer the same protection against ovarian cancer as the remaining ovary and/or tube could lead to a malignancy. Not all CPT codes separate unilateral from bilateral procedures and thus there is some uncertainty on the extent of adnexal removal with patients coded this way. Also, some hysterectomy codes are nonspecific as to inclusion of adnexal removal or not. Most notably, abdominal hysterectomy CPT codes can include patient “with or without” adnexal removal. Thus, using these codes can contribute uncertainty to the study as the true proportion of patient with adnexal removal with hysterectomy is not indicated by the code. About 30% of the procedures included in this study came from these questionable codes and the estimates for SO prevalence are likely over estimated because of their inclusion. Future studies can address these uncertainties by verifying procedures performed by complete chart review. This technique is time consuming and would limit the number of patients able to be studied, but is important for refining the exact risk estimates. The advancement of electronic medical records and more specific ICD coding will make this kind of confirmation more feasible in the future.

Additionally, because the KHCD data prior to 2004 are not available, the SO rates prior to 2004 were based on assumptions. This will certainly generate biases for the estimates. Although this study requires a range of assumptions, it is reasonable to assume the true estimate is captured within the variation of the estimates. There are no survival data available in the KHCD data, hence the 2010 US life table estimates with a 0.5% deduction were used to estimate the alive SO cases for specific prevalence date and age groups. How much bias is introduced and in which direction this bias goes is unknown. With increasing availability and reliability of health claims data, this approach will likely provide more accurate estimates in the future. The BRFSS data are limited by sampling biases, recall biases, and missing data. Overall, although we cannot provide specific rates for the SO estimates, we can conclude that the corrected rates of ovarian cancer were substantially higher when SO was taken into consideration than estimates from the standard population. 

Finally, this study should be expanded to a broader national population as there may be important differences between the population in Kentucky versus other parts of the nation.

In conclusion, this study presents an important concept for correcting the underestimation of ovarian cancer risk for women who retain their ovaries and tubes. This correction has critical implications for the calculation of screening program performance in terms of predictive value. It is also critically important to refine the epidemiological assessment of the distribution of this disease and the populations at risk so that the highest risk groups can be identified, which will improve screening programs ability to reduce mortality from ovarian cancer while reducing harm from unnecessary interventions.

## Figures and Tables

**Table 1:** Salpingo-oophorectomy (SO) cases in Kentucky Hospital Discharge Data by age group, 2004–2013.

Year	Age Group																		
	0	1–5	6–10	11–15	16–20	21–25	26–30	31–35	36–40	41–45	46–50	51–55	56–60	61–65	66–70	71–75	76–80	81 and over	Total
2004	1	1	0	6	24	179	561	966	1482	2077	1919	987	445	356	265	202	142	79	9692
2005	0	1	1	7	24	191	542	887	1342	1999	1874	935	501	323	242	179	121	80	9249
2006	0	0	0	6	24	184	536	954	1308	1798	1662	894	500	365	277	201	142	87	8938
2007	1	0	1	5	31	193	500	833	1299	1669	1625	923	574	371	233	200	119	96	8673
2008	0	0	1	6	26	172	462	769	1119	1549	1512	832	486	382	306	179	124	86	8011
2009	1	2	0	8	38	147	500	771	1123	1530	1509	851	500	436	291	182	119	89	8097
2010	0	0	0	10	32	162	454	783	1075	1327	1373	828	479	384	265	198	112	75	7557
2011	2	1	2	12	40	155	411	764	1008	1241	1288	777	473	416	310	182	72	67	7221
2012	0	0	0	6	31	130	363	699	941	1185	1205	781	488	391	274	191	94	72	6851
2013	0	0	0	9	27	122	366	726	1031	1256	1188	748	518	425	307	186	103	63	7075
Total	5	5	5	75	297	1635	4695	8152	11,728	15,631	15,155	8556	4964	3849	2770	1900	1148	794	81,364

**Table 2:** Estimated SO Prevalence in Kentucky for year 2009 and 2013, by age groups *.

Age Group	Prob. of Survival	2009			2013		
		Population	Prevalence Count	Prevalence Rate	Population	Prevalence Count	Prevalence Rate
0	0.989	27,302	1.0	0.000	26,905	0.0	0.000
1–5	0.995	138,333	2.2	0.000	135,862	1.0	0.000
6–10	0.995	137,647	4.8	0.000	138,692	3.8	0.000
11–15	0.995	137,317	23.2	0.000	139,204	32.4	0.000
16–20	0.995	146,960	128.7	0.001	138,987	134.5	0.001
21–25	0.995	139,412	689.7	0.005	154,076	596.8	0.004
26–30	0.994	143,447	2624.1	0.018	137,250	2201.0	0.016
31–35	0.994	135,456	6206.3	0.046	142,561	5680.1	0.040
36–40	0.994	145,489	11,649.2	0.080	135,750	10,662.2	0.079
41–45	0.993	150,939	19,138.6	0.127	146,947	17,331.1	0.118
46–50	0.992	164,356	27,186.7	0.165	154,584	25,180.5	0.163
51–55	0.991	159,697	31,613.2	0.198	163,369	31,226.4	0.191
56–60	0.989	142,234	32,472.1	0.228	153,709	33,146.2	0.216
61–65	0.987	118,111	32,339.0	0.274	134,958	32,887.2	0.244
66–70	0.982	92,015	31,612.5	0.344	106,051	31,852.8	0.300
71–75	0.974	71,219	30,014.6	0.421	78,893	30,093.7	0.381
76–80	0.960	58,333	27,272.4	0.468	58,703	27,192.7	0.463
81+	0.911	84,869	39,805.1	0.469	87,494	39,812.0	0.455

* Assume SO incidence prior 2004 same as the average of incidence between years 2004–2013.

**Table 3:** Estimated hysterectomy and oophorectomy prevalence based on the Kentucky BRFSS data and discharge data.

Age Group	Hysterectomy Prevalence Rate by BRFSS ^	Ratio of SO vs. Hysterectomy *	SO Prevalence Rate by BRFSS
21–25	0.000	0.902	0.000
26–30	0.024	0.723	0.018
31–35	0.076	0.665	0.051
36–40	0.120	0.649	0.078
41–45	0.196	0.719	0.141
46–50	0.256	0.879	0.225
51–55	0.379	1.010	0.383
56–60	0.415	1.033	0.429
61–65	0.461	1.021	0.471
66–70	0.513	0.988	0.507
71–75	0.517	0.963	0.498
76–80	0.532	0.900	0.479
81+	0.512	0.829	0.425

^ Estimated hysterectomy prevalence based on the KY BRFSS data, 2008–2012; * Ratio of SO vs. hysterectomy in Kentucky discharge data from year 2004 to 2013.

**Table 4:** Age adjusted rates for invasive ovary cancer in Kentucky, 2009–2013.

Type of Population Under Risk	All Ages				
	Population under Risk	*N*	Adj Rate	95% CI	
Standard Population ^	11,083,781	1403	10.73	10.16	11.32
Standard Population with Modified Age Group *	11,083,781	1403	10.73	10.17	11.32
Modified Population based on KCHD-Assumption 1 ~	9,630,865	1403	15.47	14.65	16.32
Modified Population based on KCHD-Assumption 2 ~	9,414,282	1403	16.88	15.98	17.82
Modified Population based on KCHD-Assumption 3 ~	9,847,449	1403	14.34	13.58	15.12
Modified Population based on BRFS †	9,009,436	1387	17.72	16.78	18.69

^ The standard 19 population age groups, 0, 1–4, 5–9, …, 80–84, 85+; * Use the 18 age groups in the hospital discharge data, 0, 1–5, 6–10, …, 76–80, 81+; ~ Adjusted the standard population based on the prevalence rates from the Kentucky Health Claims Data; Assumption 1: Assume incidence prior to2004 same as the average in year 2004–2013; Assumption 2: Assume incidence prior to 2004 same as the average in year 2004; Assumption 3: Assume incidence prior to 2004 same as the average in year 2012; † Adjusted the standard population based on the prevalence rates from BRFSS data.

## Supplementary Materials

The following are available online at www.mdpi.com/2075-4418/07/2/019/s1.
