# Supplementary material for: Ovarian Cancer Incidence Corrected for Oophorectomy
Source: Diagnostics (Basel). 2017 Apr 1;7(2):19. doi: 10.3390/diagnostics7020019 (PMC5489939; doi:10.3390/diagnostics7020019)
Supplement: Supplementary file 1 [file diagnostics-07-00019-s001.docx]

Supplementary Materials: Ovarian Cancer Incidence Corrected for Oophorectomy

Lauren A. Baldwin, Quan Chen, Thomas Tucker, Connie G. White, Robert Ore, and Bin Huang

**Table S1.** Estimated salpingo-oophorectomy (SO) prevalence in Kentucky for years 2009 and 2013, by age groups, assuming SO incidence prior 2004 same as in year 2004.

| **Age Group** | **Prob of Survival** | **2009** | | | **2013** | | |
| --- | --- | --- | --- | --- | --- | --- | --- |
|  |  | **Population** | **Prevalence Count** | **Prevalence Rate** | **Population** | **Prevalence Count** | **Prevalence Rate** |
| 0 | 0.989 | 27,302 | 1.0 | 0.000 | 26,905 | 0.0 | 0.000 |
| 1–5 | 0.995 | 138,333 | 2.2 | 0.000 | 135,862 | 1.0 | 0.000 |
| 6–10 | 0.995 | 137,647 | 6.2 | 0.000 | 138,692 | 3.9 | 0.000 |
| 11–15 | 0.995 | 137,317 | 25.5 | 0.000 | 139,204 | 34.2 | 0.000 |
| 16–20 | 0.995 | 146,960 | 131.0 | 0.001 | 138,987 | 136.8 | 0.001 |
| 21–25 | 0.995 | 139,412 | 681.8 | 0.005 | 154,076 | 598.4 | 0.004 |
| 26–30 | 0.994 | 143,447 | 2680.5 | 0.019 | 137,250 | 2195.3 | 0.016 |
| 31–35 | 0.994 | 135,456 | 6593.7 | 0.049 | 142,561 | 5772.4 | 0.040 |
| 36–40 | 0.994 | 145,489 | 12,598.9 | 0.087 | 135,750 | 11,138.8 | 0.082 |
| 41–45 | 0.993 | 150,939 | 21,091.0 | 0.140 | 146,947 | 18,404.3 | 0.125 |
| 46–50 | 0.992 | 164,356 | 30,863.4 | 0.188 | 154,584 | 27,356.8 | 0.177 |
| 51–55 | 0.991 | 159,697 | 37,011.5 | 0.232 | 163,369 | 35,171.1 | 0.215 |
| 56–60 | 0.989 | 142,234 | 38,603.3 | 0.271 | 153,709 | 38,604.6 | 0.251 |
| 61–65 | 0.987 | 118,111 | 38,296.1 | 0.324 | 134,958 | 38,785.2 | 0.287 |
| 66–70 | 0.982 | 92,015 | 37,118.4 | 0.403 | 106,051 | 37,426.6 | 0.353 |
| 71–75 | 0.974 | 71,219 | 35,036.3 | 0.492 | 78,893 | 35,145.1 | 0.445 |
| 76–80 | 0.960 | 58,333 | 31,729.7 | 0.544 | 58,703 | 31,642.2 | 0.539 |
| 81+ | 0.911 | 84,869 | 46,326.0 | 0.546 | 87,494 | 46,259.8 | 0.529 |

**Table S2.** Estimated SO prevalence in Kentucky for year 2009 and 2013, by age groups, assuming SO incidence prior 2004 same as in year 2012.

| **Age Group** | **Prob of Survival** | **2009** | | | **2013** | | |
| --- | --- | --- | --- | --- | --- | --- | --- |
|  |  | **Population** | **Prevalence Count** | **Prevalence Rate** | **Population** | **Prevalence Count** | **Prevalence Rate** |
| 0 | 0.989 | 27,302 | 1.0 | 0.000 | 26,905 | 0.0 | 0.000 |
| 1–5 | 0.995 | 138,333 | 2.2 | 0.000 | 135,862 | 1.0 | 0.000 |
| 6–10 | 0.995 | 137,647 | 3.3 | 0.000 | 138,692 | 3.7 | 0.000 |
| 11–15 | 0.995 | 137,317 | 20.8 | 0.000 | 139,204 | 30.6 | 0.000 |
| 16–20 | 0.995 | 146,960 | 126.4 | 0.001 | 138,987 | 132.2 | 0.001 |
| 21–25 | 0.995 | 139,412 | 697.6 | 0.005 | 154,076 | 595.2 | 0.004 |
| 26–30 | 0.994 | 143,447 | 2567.6 | 0.018 | 137,250 | 2206.7 | 0.016 |
| 31–35 | 0.994 | 135,456 | 5819.0 | 0.043 | 142,561 | 5587.9 | 0.039 |
| 36–40 | 0.994 | 145,489 | 10,699.5 | 0.074 | 135,750 | 10,185.5 | 0.075 |
| 41–45 | 0.993 | 150,939 | 17,186.1 | 0.114 | 146,947 | 16,257.8 | 0.111 |
| 46–50 | 0.992 | 164,356 | 23,510.0 | 0.143 | 154,584 | 23,004.1 | 0.149 |
| 51–55 | 0.991 | 159,697 | 26,215.0 | 0.164 | 163,369 | 27,281.6 | 0.167 |
| 56–60 | 0.989 | 142,234 | 26,340.8 | 0.185 | 153,709 | 27,687.9 | 0.180 |
| 61–65 | 0.987 | 118,111 | 26,381.9 | 0.223 | 134,958 | 26,989.3 | 0.200 |
| 66–70 | 0.982 | 92,015 | 26,106.5 | 0.284 | 106,051 | 26,279.1 | 0.248 |
| 71–75 | 0.974 | 71,219 | 24,992.9 | 0.351 | 78,893 | 25,042.4 | 0.317 |
| 76–80 | 0.960 | 58,333 | 22,815.0 | 0.391 | 58,703 | 22,743.2 | 0.387 |
| 81+ | 0.911 | 84,869 | 33,284.3 | 0.392 | 87,494 | 33,364.2 | 0.381 |

**Table S3.** Age specific rates for invasive ovary cancer in Kentucky, 2009–2013.

| **Age Groups** | **Uncorrected** | **Corrected** | | | |
| --- | --- | --- | --- | --- | --- |
|  |  | **Assumption 1** | **Assumption 2** | **Assumption 3** | **BRFSS** |
| 0 |  |  |  |  |  |
| 1–5 |  |  |  |  |  |
| 6–10 | 0.29 | 0.29 | 0.29 | 0.29 |  |
| 11–15 | 0.58 | 0.58 | 0.58 | 0.58 |  |
| 16–20 | 1.39 | 1.39 | 1.39 | 1.39 |  |
| 21–25 | 1.37 | 1.37 | 1.37 | 1.37 | 1.37 |
| 26–30 | 3.27 | 3.32 | 3.32 | 3.32 | 3.32 |
| 31–35 | 3.16 | 3.30 | 3.30 | 3.29 | 3.33 |
| 36–40 | 4.84 | 5.25 | 5.28 | 5.22 | 5.24 |
| 41–45 | 9.48 | 10.81 | 10.94 | 10.69 | 11.04 |
| 46–50 | 13.17 | 15.73 | 16.08 | 15.40 | 16.98 |
| 51–55 | 20.71 | 25.70 | 26.66 | 24.81 | 33.57 |
| 56–60 | 19.60 | 25.19 | 26.53 | 23.97 | 34.33 |
| 61–65 | 26.61 | 35.67 | 38.03 | 33.58 | 50.30 |
| 66–70 | 29.97 | 44.42 | 48.51 | 40.97 | 60.79 |
| 71–75 | 44.71 | 74.80 | 84.31 | 67.22 | 89.02 |
| 76–80 | 38.46 | 72.22 | 84.32 | 63.15 | 73.85 |
| 81 and over | 49.31 | 91.48 | 106.28 | 80.30 | 85.69 |

Uncorrected: Standard age-specific SO rates without correcting the background risk population; Assumption 1: assuming SO incidence rates prior 2004 same as the average in year 2004–2013; Assumption 2: assuming SO incidence rates prior 2004 same as the average in year 2004; Assumption 3: assuming SO incidence rates prior 2004 same as the average in year 2012; BRFSS: Based on the BRFSS data.
